# Need for expanded Candida Score for empiric antifungal use in medically critically ill patients?

**DOI:** 10.1186/s13054-019-2525-3

**Published:** 2019-07-04

**Authors:** Melanie E. Laine, Alexander H. Flannery, Breanna Moody, Melissa L. Thompson Bastin

**Affiliations:** 1 Medical Intensive Care Unit/Pulmonary, University of Kentucky HealthCare, Lexington, USA; 2 Department of Pharmacy Practice and Science, University of Kentucky College of Pharmacy, 800 Rose Street, H110, Lexington, KY 40536 USA; 3 Department of Pharmacy, Lexington VA Health Care System, 1101 Veterans Drive, Lexington, KY 40502 USA

*Candida* spp. rank among the top four nosocomial bloodstream infections in critically ill patients with up to 40% mortality despite antifungal therapy [1]. The utility of biomarkers such as β-1,3-d-glucan and procalcitonin, alone or in combination, demonstrate promise; nevertheless, bedside scoring tools are useful for guiding clinical decision-making [2]. Recent guidelines recommend the use of risk prediction tools to facilitate earlier recognition and initiation of antifungal therapy [3]. Commonly cited is the “Candida Score,” which showed sensitivity and specificity for invasive candidiasis of 81% and 74%, respectively, for scores > 2.5 [4]. This was a mixed medical-surgical intensive care unit (ICU) patient population, with only 35% of admissions for medical reasons. Thus, the application of this tool for patients with nonsurgical reasons for ICU admission may be poor. Additionally, certain components of the score, surgery and parenteral nutrition (PN), may be less applicable to the medical ICU (MICU) population. Although other predictive tools have been developed, they have not been validated prospectively like the Candida Score and carry low positive predictive values.

Based on 10 years of experience in our MICU in patients with positive blood cultures for *Candida* spp. (*n* = 139), we found that only 37% of patients had a positive Candida Score (i.e., > 2.5) (Table 1). Sixteen percent of candidemia cases scored 0, 23% scored 1, and 24% scored 2 (Fig. 1). The most common risk factors were severe sepsis/septic shock (53%) and multifocal *Candida* colonization (62%).

**Table 1:** Patient demographics

	*n* = 139
Age (mean ± SD)	53 ± 14.4
Gender (% female)	51%
Caucasian	88%
ICU LOS (median, days)	18 (8–31)
LOS prior to positive culture (median, days)	9 (3–21)
Comorbidities	
ESRD	13 (9%)
Cirrhosis	24 (17%)
Neoplasm	25 (18%)
Necrotizing pancreatitis	5 (4%)
Corticosteroids	
Recent steroid use*	29 (21%)
Cumulative steroid dose (median, milligrams^)	745 (600–1525)
Total steroid duration (median, days)	19 (12–30)
Candida colonization	
Respiratory	59 (42%)
Urine	57 (41%)
Multifocal	86 (62%)

*SD* standard deviation, *LOS* length of stay, *ESRD* end stage renal disease

*Prednisone 20 mg equivalent × 2 weeks or 30 mg equivalent × 1 week

^Prednisone equivalents

**Fig. 1:**
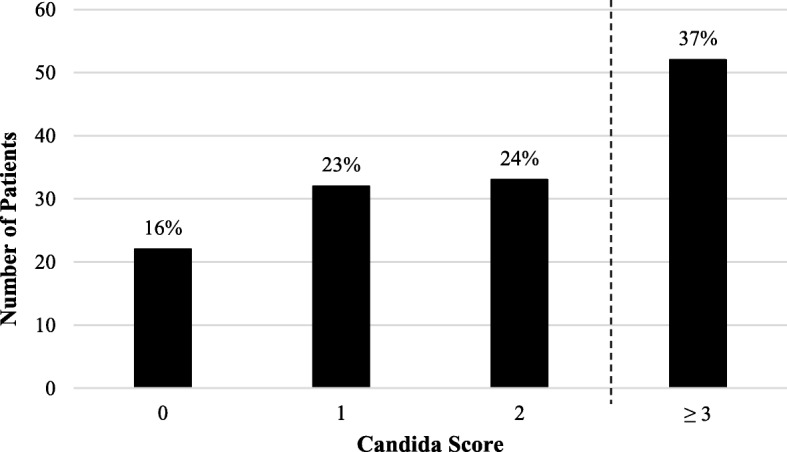
Distribution of Candida Scores

While our experience demonstrates less than half of MICU patients with candidemia meet the formal cut-off for a positive Candida Score, previous data reported an incidence of invasive candidiasis of only 2.3% with a score < 3 [5]. We note differences between our cohort and Leon et al. in those with proven infection [4]. Our patients had a much higher incidence of chronic liver disease (17% versus 2%) and more end-stage renal disease (9% versus 4.1%). Conversely, PN use was notably higher in the Leon study (87.6% versus 6%), along with recent surgery (52.6% versus 22%), as compared to our patients. Severe sepsis and multifocal *Candida* colonization were comparable, suggesting other risk factors may be present in the MICU population not captured by the Candida Score. Expanded scoring criteria is necessary to more accurately identify critically ill patients who warrant empiric antifungal therapy, and prospective studies evaluating additional risk factors and the role of non-culture diagnostics are needed.

## Data Availability

The datasets used and/or analyzed during the current study are available from the corresponding author on reasonable request.
